# Engineering DNA-based cytoskeletons for synthetic cells

**DOI:** 10.1098/rsfs.2023.0028

**Published:** 2023-08-11

**Authors:** Kevin Jahnke, Kerstin Göpfrich

**Affiliations:** ^1^ Biophysical Engineering Group, Max Planck Institute for Medical Research, Jahnstraße 29, 69120 Heidelberg, Germany; ^2^ Department of Physics and Astronomy, Heidelberg University, 69120 Heidelberg, Germany; ^3^ Center for Molecular Biology (ZMBH), Heidelberg University, Im Neuenheimer Feld 329, 69120 Heidelberg, Germany

**Keywords:** synthetic cell, bottom-up synthetic biology, cytoskeleton, DNA nanotechnology, giant unilamellar lipid vesicle, DNA origami

## Abstract

The development and bottom-up assembly of synthetic cells with a functional cytoskeleton sets a major milestone to understand cell mechanics and to develop man-made machines on the nano- and microscale. However, natural cytoskeletal components can be difficult to purify, deliberately engineer and reconstitute within synthetic cells which therefore limits the realization of multifaceted functions of modern cytoskeletons in synthetic cells. Here, we review recent progress in the development of synthetic cytoskeletons made from deoxyribonucleic acid (DNA) as a complementary strategy. In particular, we explore the capabilities and limitations of DNA cytoskeletons to mimic functions of natural cystoskeletons like reversible assembly, cargo transport, force generation, mechanical support and guided polymerization. With recent examples, we showcase the power of rationally designed DNA cytoskeletons for bottom-up assembled synthetic cells as fully engineerable entities. Nevertheless, the realization of dynamic instability, self-replication and genetic encoding as well as contractile force generating motors remains a fruitful challenge for the complete integration of multifunctional DNA-based cytoskeletons into synthetic cells.

## 1. Introduction

Bottom-up synthetic biology cultivates an engineering approach to assemble the first synthetic cell from molecular building blocks. An essential component of every modern eukaryotic cell is the cytoskeleton because it drives cell motility, stability and intracellular transport, among other functions [1]. In this regard, major efforts have been made concerning the reconstitution of natural cytoskeletons and their auxiliary proteins into cell-sized confinement [2]. However, protein purification and functional reconstitution can be challenging and the deliberate engineering of proteins to implement specific functions is only at the verge of being considered for practical applications. DNA nanotechnology offers a chemically functionalizable, programmable and versatile tool [3]. In addition, it is especially exciting to build a synthetic cell from completely synthetic building blocks and to test our knowledge on natural cytoskeletons by comparing it to rationally engineered mimics thereof. Here, we will review current progress in the design and assembly of DNA-based cytoskeletons and discuss their use for the engineering of synthetic cells with a synthetic cytoskeleton.

DNA nanotechnology allows one to programmably assemble DNA strands into higher-order structures using the complementary base pairing rules. These structures can be classified into several basic building blocks, including DNA linkers, Y-motifs, DNA tiles and DNA origami, which again can be assembled into larger architectures like DNA lattices and DNA nanotubes in a hierarchical manner [4]. The potential to engineer functional parts using these programmable and biocompatible building blocks has been especially interesting for use in bottom-up synthetic biology [3]. Examples include, among others, DNA-based scramblases [5], ion channels [6] and porins [7,8], cell–cell linkers [9,10], assembly platforms [11,12] or adhesion sites [13]. In recent years, DNA-based mimics of cytoskeletal elements and cortices have become a particularly prominent example.

**Figure 1.**
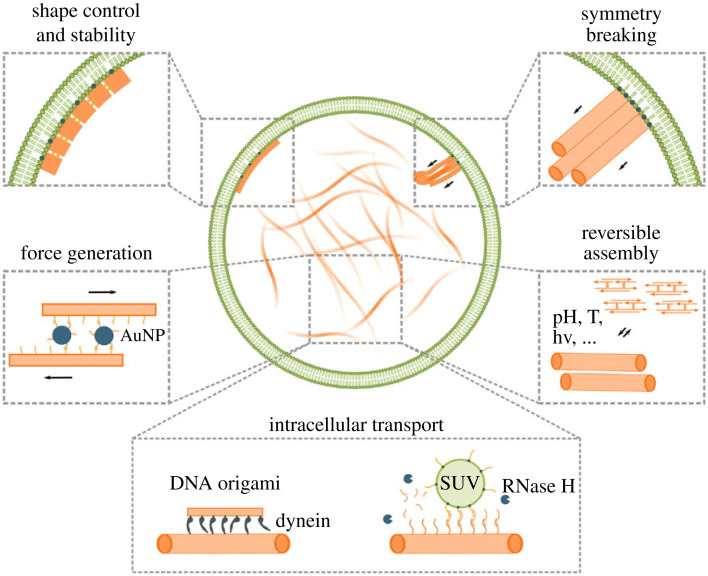
Engineering synthetic cytoskeletons with DNA nanotechnology. Engineerable functions include (i) mechanical control and morphology control, (ii) reversible assembly, (iii) symmetry breaking, (iv) intracellular transport and (v) force generation.

The challenge towards the assembly of a DNA-based cytoskeleton is that it should not just perform one function but execute several different tasks on demand. DNA nanotubes are an important early type of DNA nanostructures [14] and visually resemble cytoskeletal elements. At the same time, the advances in DNA nanotechnology go hand-in-hand with the establishment of reliable and easy-to-use encapsulation techniques into cell-sized compartments by means of microfluidics or other techniques [15,16]. Giant unilamellar vesicles (GUVs) are a popular compartment choice for harbouring synthetic cellular components, as they mimic the lipid bilayer membrane of natural cells. Various strategies exist for GUV formation, which have been developed further to optimize encapsulation properties, e.g. octanol-assisted liposome assembly (OLA) [17], continuous droplet interface crossing encapsulation (cDICE) [18,19] or the droplet-stabilized GUV formation method (dsGUVs) [20,21]. Here, we want to review the recent progress and development of DNA nanostructures for their use as a DNA cytoskeleton within cell-sized confinement and in particular GUVs. We will focus on five different key functions of natural cytoskeletons and how these can be mimicked or potentially even surpassed by DNA cytoskeletons. The main functionalities include (i) mechanical control and morphology control, (ii) reversible assembly, (iii) symmetry breaking, (iv) intracellular transport and (v) force generation as illustrated in figure 1.

## 2. Functional DNA cytoskeletons

The key challenge for building DNA cytoskeletons is to reach the same sophistication as natural cytoskeletal elements using DNA as an entirely different building material with distinct chemical and physical properties. As a starting point, we first explore already existing DNA nanostructures that mimic one specific key property of natural cytoskeletons. Then, we discuss how they could be merged into one multifunctional synthetic cytoskeleton for a synthetic cell and which elements are still missing, providing exciting directions for future research.

### 2.1. Shape control and stability

Natural cells are mechanically stable in order to resist stress from the environment. However, their mechanical properties strongly depend on the cell type, i.e. a red blood cell is more deformable than an epithelial cell [22]. This goes hand in hand with the degree to which the cellular morphology is determining the cell’s function. The viscoelastic properties of cells are governed by their cytoskeletal cortex, which forms a crosslinked network underneath the cell’s plasma membrane. On the contrary, the rigidity of a GUV as synthetic cell container is only tuneable to a very limited extent by the choice of lipids, e.g. by changing their fatty acid tail length, saturation, charge or headgroup modifications [23]. In order to change the mechanical properties and the local morphology of GUVs, versatile DNA nanostructures have been developed.

**Figure 2.**
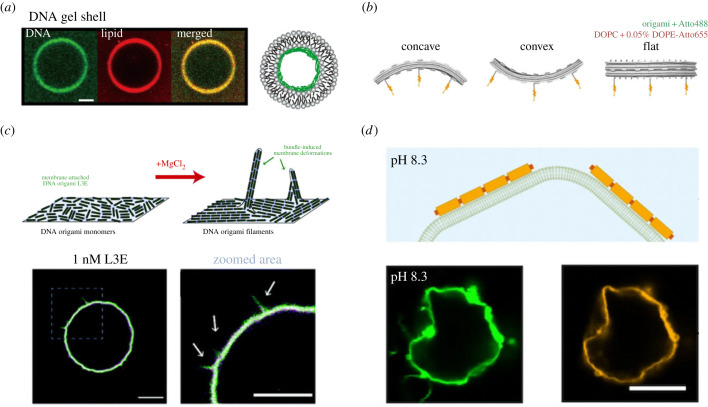
DNA nanostructures for mechanical support and morphology control of giant unilamellar vesicles. (*a*) DNA Y-motifs polymerizing into lattices that stabilize GUVs from the inside [24]. Scale bar: 10 μm. (*b*) Flat and curved DNA origami structures to scaffold lipid membranes [25]. (*c*) DNA origami rods lead to spike-like membrane protrusions mediated via divalent ions [26]. Scale bar: 5 μm. (*d*) pH-sensitive deformation of GUVs via polymerized DNA origami [27]. Scale bar: 10 μm.

In particular, several studies have shown that GUVs can be mechanically stabilized by crosslinking DNA nanostructures on the membrane surface. One way to achieve an increasing mechanical stability of lipid vesicles is to use so-called DNA Y-motifs or triskelia that can hybridize with one another to form micrometre-scale lattices. These lattices have been bound to lipid membranes via charge-mediated interactions with positively charged lipids [24]. The gel-like shell outside or inside GUVs, likely composed of multiple layers of DNA, was shown to endow GUVs with higher mechanical stability leading to higher survival rates after osmotic shock [24] (figure 2*a*). A different approach is the targeted functionalization of individual DNA strands with hydrophobic moieties that incorporate in the lipid bilayer of GUVs [28] or natural cells [29]. These hydrophobic anchors include tocopherol, palmitoyl, porphyrin or, most commonly, cholesterol [29]. By modifying the DNA triskelia with cholesterol, a similar enhancement of vesicle stability has been used to coat the lipid membrane inspired by the protein clathrin [30]. The clathrin-inspired DNA lattice coating of vesicles can also be used for triggered cargo release based on toehold-mediated strand displacement reactions [31]. Whether the attachment of DNA nanostructures to lipid bilayers requires hydrophobic tags or can be achieved by electrostatic interactions only crucially depends on the buffer condition, the lipid composition as well as the DNA structures themselves. In particular at low concentrations of divalent ions, hydrophobic tags are often needed for accurate attachment [32].

DNA origami has often been employed as an alternative strategy for the deformation and scaffolding of lipid membranes. DNA origami depends on the folding of a long scaffold strand with hundreds of short staple strands and allows the programmable formation of tens of nanometre- to micrometre-sized structures [33]. In the context of scaffolding lipid membranes, flat DNA origami structures as well as designs with positive or negative curvature have been used (figure 2*b*) [25]. Based on the DNA origami curvature, the attachment to GUVs resulted in outward tubules (concave), invaginations (convex) or no deformation (no curvature). This concept has also been expanded on DNA origami nanosprings with highly curved geometries that induce vesicle tubulation [34]. Note that membrane curvature is introduced in any case when polymers like DNA are absorbed on the membrane [35]; curved DNA origami is not a prerequisite. Instead of DNA origami curvature, the GUV deformation can also be controlled by the ionic strength of the solution, which regulates the DNA origami diffusion on the lipid membrane [36]. This effect was exploited to create spike-like membrane protrusions depending on the magnesium-ion concentration with DNA origami rods (figure 2*c*) [26,37]. In addition to curvature and ionic strength, a third approach for GUV deformation relies on DNA origami oligo- and polymerization into bigger clusters, which in turn also have a higher persistence length and lower diffusion [38]. As an additional trigger, the deformation of GUVs via DNA origami polymerization can also be combined with pH-sensitive triplex motifs, which makes the DNA origami attach in a pH-dependent manner (figure 2*d*) [27].

Finally, the scope of membrane deformation has also been expanded on the use of DNA four-arm motifs [39] and DNA nanotubes to control GUV morphology [40,41].

### 2.2. Reversible assembly

Many cytoskeletal functions rely on the reversible assembly of actin, intermediate filaments and microtubules. Most prominently, microtubules polymerize in the presence of GTP leading to the property of dynamic instability, where the microtubule ends grow and shrink on their ends depending on GTP hydrolysis [42]. To implement similar features and in particular the stimuli-responsive reversible assembly of DNA nanostructures, several groups used DNA nanotubes as model system.

**Figure 3.**
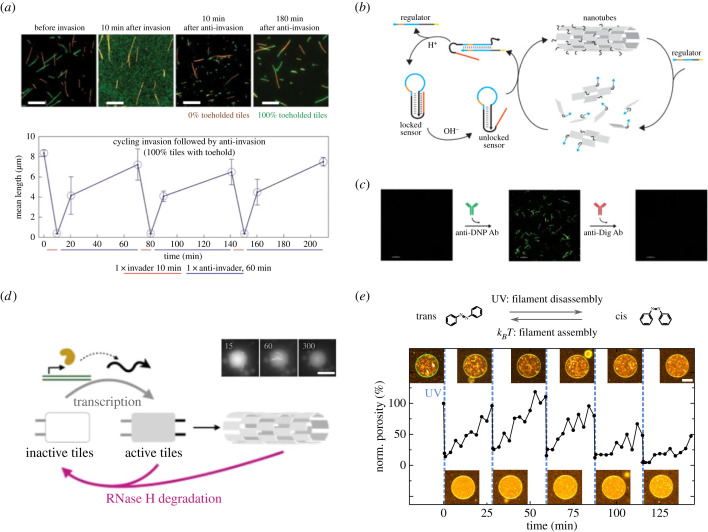
Reversible assembly of DNA nanotubes. Reversible DNA nanotube assembly can be controlled via toehold-mediated strand displacement (*a*) [43], via pH by using a triplex motif (*b*) [44], via antibodies by using antigen-functionalized DNA (*c*), via enzymes (*d*) [45] or via light (*e*) [40]. Scale bars: 10 μm (*a*,*e*), 20 μm (*d*), 5 μm (*c*).

DNA nanotubes are made up of individual DNA tiles that can hybridize with one another due to complementary overhangs [14]. These serve as modification sites for inducing the assembly and disassembly of DNA nanotubes. One approach towards is this aim is using toehold-mediated strand displacement (figure 3*a*). Upon addition of an invader strand that binds to the DNA tile overhang, they are inactivated leading to DNA nanotube disassembly [43]. Their reassembly can be induced by addition of an anti-invader strand that binds the invader strand and reactivates the DNA tile. This mechanism can be repeated several times. However, it requires the continuous addition of DNA strands in higher concentrations making it undesirable for many assembly–disassembly cycles. As an alternative trigger for more assembly–disassembly cycles of DNA nanotubes pH can be used (figure 3*b*). This approach makes use of a so-called triplex motif that forms pH-sensitive Hogsteen interactions and can thus bind a partially complementary strand at low pH values [44]. This, in turn, leads to the disassembly of DNA nanotubes. After changing the pH again, the DNA triplex closes and initiates DNA nanotube reassembly. Similarly, antibodies can be used for the reversible assembly by binding antigen-functionalized DNA strands (figure 3*c*) [46]. Going one step further, DNA nanotubes have not only been assembled reversibly but also reconstituted within cell-sized water-in-oil droplets [45,47]. The assembly within confinement was coupled to transcription of a DNA template leading to DNA nanotube assembly and simultaneous disassembly of DNA nanotubes due to RNase H-mediated degradation (figure 3*d*) [45]. RNAse H has also been used to reorganize DNA nanotubes into defined homo- and block copolymers [48]. To closer mimic natural cytoskeletons, however, also physiological triggers like ATP [47,49] or proteins like nucleolin [47] can be employed to induce DNA nanotube assembly–disassembly cycles. This mechanism uses DNA aptamers for DNA filament assembly and can, in principle, also be expanded to other DNA aptamers. Finally, as a non-invasive trigger for the reversible assembly of DNA nanotubes also light has been shown to induce DNA nanotube disassembly–assembly cycles within GUVs (figure 3*e*) [40].

Transferring similar concepts apart from DNA nanotubes, only recently, massive strand displacement has been used to induce shape changes of DNA origami that can fold and evolve into different forms upon stimulus presence [50].

### 2.3. Symmetry breaking

**Figure 4.**
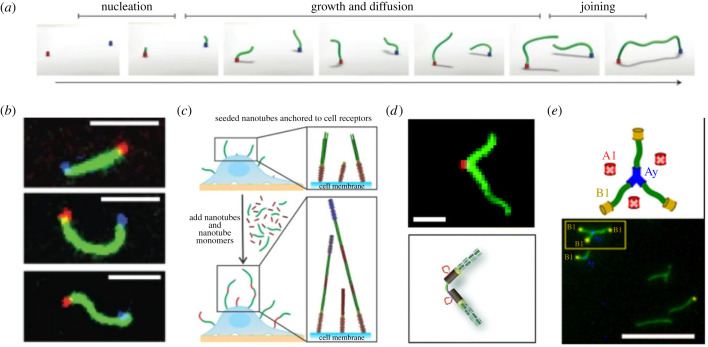
Asymmetric growth of DNA nanotubes. (*a*) Schematic illustration of DNA nanotubes growing from a DNA origami seed [55]. (*b*) Asymmetrically grown DNA nanotubes connect molecular landmarks [55]. (*c*) DNA nanotubes growing on the cell surface acting as shear force sensors [56]. (*d*) DNA nanotubes growing in angled directions determined by the DNA origami seed conformation [57]. (*e*) Hierarchical growth of DNA nanotubes via terminus inactivation [58]. Scale bars: 5 μm (*b*,*d*), 10 μm (*e*).

Symmetry breaking within biological systems is essential for cell motility and polarity [51]. Both of these features of natural cells are tightly connected to the cytoskeleton. Therefore, symmetry breaking behaviour is also desirable for synthetic reconstituted DNA cytoskeletons. In order to achieve this asymmetry, DNA origami and DNA nanotubes have been combined within one DNA nanostructure. Here, a hollow cylindrical DNA origami serves as a polymerization seed for DNA nanotubes [52,53]. Using this principle, each end of a DNA nanotube can be addressed separately and in an asymmetric way [54]. As a first proof-of-principle this allowed the end-to-end joining of DNA nanotubes that grew from two different DNA origami seeds to connect molecular landmarks (figure 4*a*,*b*) [55]. The individually polymerizing DNA nanotubes connect over time after reaching a certain length and diffusion time. As a first application DNA origami-seeded DNA nanotubes have been attached on the outer membrane of natural cells to measure the shear flow of the surrounding solution (figure 4*c*) [56]. Inserting the DNA origami seed/DNA nanotube constructs into the membrane of lipid vesicles can even lead to influx of ions and small molecules through the DNA nanotube [59,60]. Recently, DNA origami seed/DNA nanotube conjugates have also been used to transduce mechanical signals into GUVs [61]. In this work, the externally induced clustering of transmembrane DNA origami seeds has been used to induce the rearrangement of DNA nanotubes inside GUVs.

Showcasing the unique programmability of DNA origami, one can even go one step further and design seeds that allow two (figure 4*d*)[57] or even three (figure 4*e*) [58] DNA nanotubes to grow in a predefined direction. In analogy to natural cytoskeletons, these could act as centrioles or cytoskeletal binding proteins that govern and direct the shape of the cytoskeletal filaments.

### 2.4. Intracellular transport

Cytoskeletal filaments transport cargo and in particular vesicles within all eukaryotic cells. Owing to motion of molecular motors they serve as filament tracks to direct the transport of cargo to organelles. [1]. Despite the fact that the implementation of cargo transport with DNA nanotechnology poses great challenges due to the absence of motor proteins, there exist many different strategies to move cargo along DNA nanostructures.

**Figure 5.**
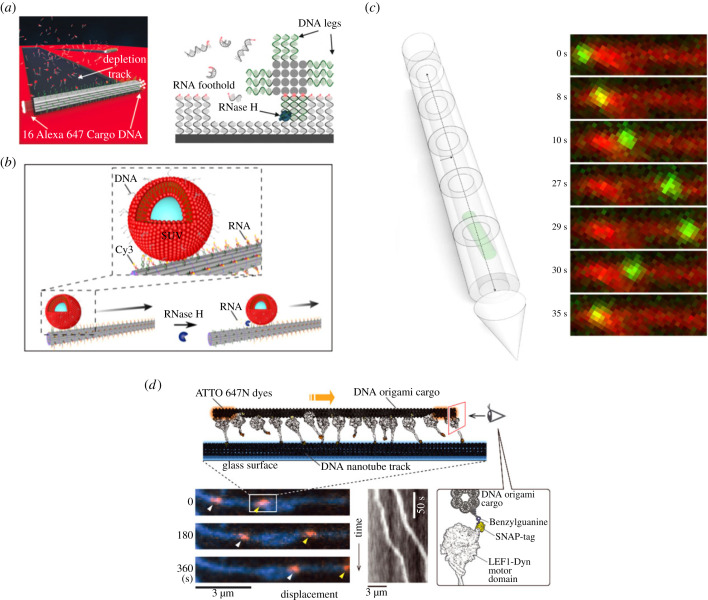
Cargo transport with DNA nanotechnology. (*a*) DNA origami roll along a surface using an RNase H-mediated burnt-bridge mechanism [70]. (*b*) The same mechanism can be used for the autonomous transport of SUVs along DNA nanotubes [47]. (*c*) DNA origami transport system using electric fields [71]. (*d*) Engineered protein motors on DNA nanotubes move DNA origami cargo [72].

An easy-to-implement strategy is the use of toehold-mediated strand displacement to engineer DNA walkers [62,63]. A major drawback from mechanisms based on strand displacement reactions is the slow timescale of the invasion, i.e. the displacement of one strand by another with higher binding affinity, which is typically of the order of minutes. Current state-of-the-art strand displacement reactions yield rate constants of the order of 1 min^−1^, which limits the transport speed. However, by changing the design of the DNA walker to allow a cartwheeling motion transport velocities of the order of 100 nm min^−1^ can be obtained [51]. Still, the size of the cargo is limited to small molecules [64] or gold nanoparticles [65] which can be attached to the single-stranded DNA. A different mechanism uses the hydrolytic activity of RNase H, which selectively cleaves RNA–DNA duplexes and induces a burnt-bridge motion of silica particles [66,67], gold nanoparticles [68], glass beads [69] or DNA origami (figure 5*a*) [70]. The achieved velocities of the RNase H-mediated mechanism are between 40 nm min^−1^ and 2 μm min^−1^. In order to apply this strategy for synthetic DNA cytoskeletons, SUVs were attached to DNA nanotubes and their motion along the filament track induced via RNase H (figure 5*b*) [47]. In contrast to the use of an enzyme to generate cargo transport, several studies have also used electric fields as transport-inducing stimulus. Electric fields are a suitable trigger for the manipulation of DNA due to its negative charge. The transport can be directed in either a rotational motion using a robotic DNA origami arm [73] or translational motion by designing a tubular DNA origami transport system (figure 5*c*) [71]. While this mechanism allows about five times faster transport than DNA walker-based strategies, the transport distance is limited by the length of the DNA origami tube, which currently is about 3 μm. Additionally, the velocity of DNA-based motors is still slow compared with natural motors which move with velocities of the order of 1 μm s^−1^. An alternative to the presented approaches is the use of natural motors to move on DNA nanotubes [74]. By modifying the natural motor protein dynein with DNA-binding domains and the DNA nanotube with recognition sites, the protein motors can move DNA nanotubes reminiscent to gliding assays of actin filaments or microtubules (figure 5*d*) [72]. Moreover, by attaching the engineered motors to DNA origami, the DNA origami can be transported with velocities of 20 nm s^−1^ along the DNA nanotube track. By implementing the previously described branching of DNA nanotubes (figure 2*e*) the cargo can also be sorted based on the choice of the DNA binding site. However, the use of proteins poses its own challenges because they are difficult to purify, engineer for specific functions and combine with other functional sets of proteins.

All in all, these versatile strategies to induce cargo transport along DNA nanostructures make up a large toolbox for further studies within the confinement of GUVs.

### 2.5. Force generation

**Figure 6.**
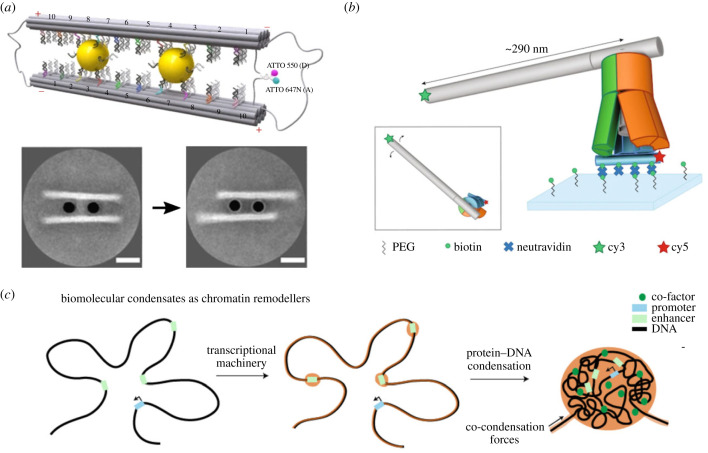
Force generation via DNA nanostructures. (*a*) Gold nanoparticles induce sliding of DNA origami filaments against each other using toehold-mediated strand displacement [77]. Scale bar: 20 nm. (*b*) Brownian motion induces the rotary motion of a 10 helix bundle arm [78]. (*c*) Protein–DNA condensation pulls non-condensed DNA [79].

Cytoskeletal filaments can exert forces within natural cells or even cell communities based on their contractility in the presence of motor proteins like myosin II. The quest for DNA nanomachines that can exert similar forces on DNA nanostructures has resulted in quite a few DNA-based applications towards this aim [75,76]. By functionalizing gold nanoparticles with single-stranded DNA and attaching them to two DNA origami filaments these can slide against each other using toehold-mediated strand displacement (figure 6*a*) [77,80]. Similarly, gold nanoparticles can also rotate DNA origami structures against each other [81–84]. Interestingly, this transition can even be triggered with an external stimulus like light based on the photoswitching properties of azobenzene incorporated into single-stranded DNA [82]. Towards the engineering of rotary DNA motors that mimic the working principle of the F_1_F_0_-ATP synthase DNA origami levers have been attached to a mulitlayer DNA origami base [85–87]. Within these, the lever that has a length of up to 290 nm can freely rotate (figure 6*b*) [78]. Moreover, the lever rotation can also be coupled to mechanical transitions of the DNA origami base. Similarly, also other DNA origami nanostructures can be reconfigured based on DNA origami shape complementarity [88]. A mechanism that does not rely on DNA origami to generate forces with DNA is the condensation of DNA and proteins into liquid-like condensates. There, DNA condensation exerts forces that drive the growth of the protein–DNA condensate by pulling on non-condensed DNA (figure 6*c*) [79]. Another mechanism that does not rely on DNA origami is the formation and contraction of DNA rings via synthetic peptides [89]. In this work, DNA-binding peptides induce the crosslinking and sliding of DNA nanotubes along one another to induce ring contraction. Mechanisms involving the formation of DNA rings are particularly interesting because they are the most reminiscent of contractile forces leading to the division of natural cells.

## 3. Conclusion and outlook

The development of more and more complex synthetic cells from the bottom up has deepened our understanding of natural cells. A major focus has been the engineering and reconstitution of natural cytoskeletons into cell-sized compartments. Recent advances of DNA nanotechnology in designing programmable micrometre-sized structures that can be interfaced with GUVs open up a new path towards entirely synthetic cytoskeletons for synthetic cells, engineered from nucleic acids rather than proteins. Our continuously increasing understanding of DNA as a building block adds to the comparably easy tuneability of biophysically relevant parameters with DNA. These include size, shape, diameter, charge, functionalization, persistence and rigidity, which can be easily engineered and altered with DNA nanotechnology. Additionally, the use of DNA nanotechnology is straightforward by making use of the large toolbox of versatile structures and corresponding libraries [90].

These advancements have led to different solutions to establish the versatile functions of cytoskeletons with DNA like morphology control, reversible assembly, symmetry breaking, intracellular transport and force generation. As next challenges we identify (i) the incorporation of all these functionalities into a single DNA nanostructure, (ii) the incorporation of these multifunctional cytoskeletons into GUVs, (iii) mechanisms that allow the micrometre-sized contraction of DNA nanostructures and (iv) catalytic activity and self-regeneration.

Even though a key advantage of DNA nanotechnology is the amount of different nanostructures that perform different functions [4], cytoskeletons are multifunctional. This implies that synthetic DNA cytoskeletons should in principle also be made from a single DNA unit with engineered multifunctionality. Polymerizing Y-motifs are some of the easiest DNA nanostructures but albeit their easy handling and incorporation into GUVs also the least complex ones which poses obstacles for, for example, symmetry breaking or intracellular transport. On the other hand, DNA origami are the most complex DNA structures facing opposite problems since they can be engineered for a multitude of different purposes but often suffer from specialized annealing protocols or low concentrations after purification. Therefore, we identify DNA nanotubes, or variants thereof that are not only based on canonical basepairing, as the most promising candidate for synthetic DNA cytoskeletons. Next to their visual resemblance of microtubules they can be encapsulated at high concentrations, form micrometre-sized filaments and have been widely used for different purposes that relate to cytoskeletal function. Additionally, they can also be combined with DNA origami structures to form DNA nanotube/origami hybrids.

The next crucial step towards synthetic cytoskeletons for synthetic cells is the implementation of these into GUVs. Whereas all of the discussed cytoskeletal functions have been achieved in bulk, only some have been realized inside the confinement of lipid vesicles (table 1). Therefore, this poses another challenge for future work in this direction. However, with the many recently emerged GUV formation techniques, it seems reasonable that the amount of reconstituted DNA nanostructures inside GUVs will increase greatly over the next few years.

**Table 1.** Cytoskeletal DNA structures for synthetic cells.

function	out of GUVs	in GUVs	DNA nanostructure	natural counterpart	references
mechanical stability	yes	yes	Y-motif	actin	[24,30,31]
morphology control	yes	yes	DNA origami, DNA nanotubes	BAR proteins	[25–27,38,40,91]
reversible assembly	yes	yes	DNA nanotubes	actin, microtubules, IF	[40,43–45]
intracellular transport	yes	no	DNA nanotubes	microtubules	[47,72,74]
force generation	yes	no	DNA origami, ssDNA	myosin, kinesin, dynein	[77,78]

After having established the reconstitution of multifunctional synthetic DNA cytoskeletons into GUVs, it will be essential to improve the current strength of forces and transport velocities that can be generated by DNA cytoskeletons. As cytoskeletal forces drive cell motility by balancing filament polymerization and contraction it will be an important step for the future success of DNA cytoskeletons to achieve the active stimuli-responsive remodelling of the GUV membrane from the inside. Moreover, this mechanism can be directly related to the division of GUVs as synthetic cell compartments, e.g. by engineering DNA rings that can contract and induce vesicle fission.

All in all, the development of synthetic DNA-based cytoskeletons for synthetic cells with unique multifunctionality and straightforward tuneability will be an exciting step in the bottom-up assembly of truly synthetic cells from molecular building blocks. Thereby, DNA cytoskeletons will not only advance our knowledge in DNA nanotechnology but also serve as well-characterized model systems to understand natural cytoskeletons. Along this way, they allow the assembly of rationally engineered complex synthetic cells with applications in biomedicine, cellular biophysics and synthetic biology.

## Data Availability

This article has no additional data.

## References

[1] Alberts B, Johnson A, Lewis J, Morgan D, Raff M, Roberts K, Walter P. 2017 Molecular biology of the cell, ch. 16. New York, NY: W. W. Norton & Company.

[2] Bashirzadeh Y, Liu AP. 2019 Encapsulation of the cytoskeleton: towards mimicking the mechanics of a cell. Soft Matter **15**, 8425-8436. (10.1039/C9SM01669D) 31621750

[3] Göpfrich K, Platzman I, Spatz JP. 2018 Mastering complexity: towards bottom-up construction of multifunctional eukaryotic synthetic cells. Trends Biotechnol. **36**, 938-951. (10.1016/j.tibtech.2018.03.008) 29685820 PMC6100601

[4] Seeman NC, Sleiman HF. 2017 DNA nanotechnology. Nat. Rev. Mater. **3**, 17068. (10.1038/natrevmats.2017.68)

[5] Ohmann A, Li C-Y, Maffeo C, Nahas KA, Baumann KN, Göpfrich K, Yoo J, Keyser UF, Aksimentiev A. 2018 A synthetic enzyme built from DNA flips 10^7^ lipids per second in biological membranes. Nat. Commun. **9**, 2426. (10.1038/s41467-018-04821-5) 29930243 PMC6013447

[6] Langecker M, Arnaut V, Martin TG, List J, Renner S, Mayer M, Dietz H, Simmel FC. 2012 Synthetic lipid membrane channels formed by designed DNA nanostructures. Science **338**, 932-936. (10.1126/science.1225624) 23161995 PMC3716461

[7] Göpfrich K et al. 2016 Large-conductance transmembrane porin made from DNA origami. ACS Nano **10**, 8207-8214. (10.1021/acsnano.6b03759) 27504755 PMC5043419

[8] Fragasso A, De Franceschi N, Stömmer P, Van Der Sluis EO, Dietz H, Dekker C. 2021 Reconstitution of ultrawide DNA origami pores in liposomes for transmembrane transport of macromolecules. ACS Nano **15**, 12 768-12 779. (10.1021/acsnano.1c01669) PMC8388114 34170119

[9] Schoenit A, Giudice CL, Hahnen N, Ollech D, Jahnke K, Göpfrich K, Cavalcanti-Adam EA. 2021 Tuning epithelial cell–cell adhesion and collective dynamics with functional DNA-E-cadherin hybrid linkers. Nano Lett. **22**, 302-310. (10.1021/acs.nanolett.1c03780) 34939414 PMC8759084

[10] Hoffecker IT, Arima Y, Iwata H. 2019 Tuning intercellular adhesion with membrane-anchored oligonucleotides. J. R. Soc. Interface **16**, 20190299. (10.1098/rsif.2019.0299) 31662069 PMC6833338

[11] Meng W et al. 2016 An autonomous molecular assembler for programmable chemical synthesis. Nat. Chem. **8**, 542-548. (10.1038/nchem.2495) 27219697

[12] Gu H, Chao J, Xiao S-J, Seeman NC. 2010 A proximity-based programmable DNA nanoscale assembly line. Nature **465**, 202-205. (10.1038/nature09026) 20463734 PMC2872101

[13] Parolini L, Kotar J, Michele LD, Mognetti BM. 2016 Controlling self-assembly kinetics of DNA-functionalized liposomes using toehold exchange mechanism. ACS Nano **10**, 2392-2398. (10.1021/acsnano.5b07201) 26845414

[14] Rothemund PWK, Ekani-Nkodo A, Papadakis N, Kumar A, Fygenson DK, Winfree E. 2004 Design and characterization of programmable DNA nanotubes. J. Am. Chem. Soc. **126**, 16 344-16 352. (10.1021/ja044319l) 15600335

[15] Shang L, Cheng Y, Zhao Y. 2017 Emerging droplet microfluidics. Chem. Rev. **117**, 7964-8040. (10.1021/acs.chemrev.6b00848) 28537383

[16] Dimova R, Marques CM (eds). 2019 The giant vesicle book. Boca Raton, FL: CRC Press.

[17] Deshpande S, Caspi Y, Meijering AEC, Dekker C. 2016 Octanol-assisted liposome assembly on chip. Nat. Commun. **7**, 10447. (10.1038/ncomms10447) 26794442 PMC4735860

[18] Abkarian M, Loiseau E, Massiera G. 2011 Continuous droplet interface crossing encapsulation (cDICE) for high throughput monodisperse vesicle design. Soft Matter **7**, 4610. (10.1039/c1sm05239j)

[19] de Cauter LV et al. 2021 Optimized cDICE for efficient reconstitution of biological systems in giant unilamellar vesicles. ACS Synth. Biol. **10**, 1690-1702. (10.1021/acssynbio.1c00068) 34185516 PMC8291763

[20] Weiss M et al. 2017 Sequential bottom-up assembly of mechanically stabilized synthetic cells by microfluidics. Nat. Mater. **17**, 89-96. (10.1038/nmat5005) 29035355

[21] Göpfrich K, Haller B, Staufer O, Dreher Y, Mersdorf U, Platzman I, Spatz JP. 2019 One-pot assembly of complex giant unilamellar vesicle-based synthetic cells. ACS Synth. Biol. **8**, 937-947. (10.1021/acssynbio.9b00034) 31042361 PMC6528161

[22] Otto O et al. 2015 Real-time deformability cytometry: on-the-fly cell mechanical phenotyping. Nat. Methods **12**, 199-202. (10.1038/nmeth.3281) 25643151

[23] Dimova R. 2014 Recent developments in the field of bending rigidity measurements on membranes. Adv. Colloid Interface Sci. **208**, 225-234. (10.1016/j.cis.2014.03.003) 24666592

[24] Kurokawa C et al. 2017 DNA cytoskeleton for stabilizing artificial cells. Proc. Natl Acad. Sci. USA **114**, 7228-7233. (10.1073/pnas.1702208114) 28652345 PMC5514726

[25] Franquelim HG, Khmelinskaia A, Sobczak J-P, Dietz H, Schwille P. 2018 Membrane sculpting by curved DNA origami scaffolds. Nat. Commun. **9**, 811. (10.1038/s41467-018-03198-9) 29476101 PMC5824810

[26] Franquelim HG, Dietz H, Schwille P. 2021 Reversible membrane deformations by straight DNA origami filaments. Soft Matter **17**, 276-287. (10.1039/D0SM00150C) 32406895

[27] Jahnke K et al. 2021 Proton gradients from light-harvesting *E. coli* control DNA assemblies for synthetic cells. Nat. Commun. **12**, 3967. (10.1038/s41467-021-24103-x) 34172734 PMC8233306

[28] Shen Q, Grome MW, Yang Y, Lin C. 2019 Engineering lipid membranes with programmable DNA nanostructures. Adv. Biosyst. **4**, 1900215. (10.1002/adbi.201900215) 31934608 PMC6957268

[29] Schoenit A, Cavalcanti-Adam EA, Göpfrich K. 2021 Functionalization of cellular membranes with DNA nanotechnology. Trends Biotechnol. **39**, 1208-1220. (10.1016/j.tibtech.2021.02.002) 33722382

[30] Baumann KN, Piantanida L, García-Nafría J, Sobota D, Voïtchovsky K, Knowles TPJ, Hernández-Ainsa S. 2020 Coating and stabilization of liposomes by clathrin-inspired DNA self-assembly. ACS Nano **14**, 2316-2323. (10.1021/acsnano.9b09453) 31976654 PMC7302506

[31] Baumann KN, Schröder T, Ciryam PS, Morzy D, Tinnefeld P, Knowles TPJ, Hernández-Ainsa S. 2022 DNA-liposome hybrid carriers for triggered cargo release. ACS Appl. Bio Mater. **5**, 3713-3721. (10.1021/acsabm.2c00225) PMC9382633 35838663

[32] Morzy D, Rubio-Sánchez R, Joshi H, Aksimentiev A, Michele LD, Keyser UF. 2021 Cations regulate membrane attachment and functionality of DNA nanostructures. J. Am. Chem. Soc. **143**, 7358-7367. (10.1021/jacs.1c00166) 33961742 PMC8154537

[33] Dey S et al. 2021 DNA origami. Nat. Rev. Methods Primers **1**, 13. (10.1038/s43586-020-00009-8)

[34] Grome MW, Zhang Z, Pincet F, Lin C. 2018 Vesicle tubulation with self-assembling DNA nanosprings. Angew. Chem. Int. Ed. **57**, 5330-5334. (10.1002/anie.201800141) PMC5924453 29575478

[35] Nikolov V, Lipowsky R, Dimova R. 2007 Behavior of giant vesicles with anchored DNA molecules. Biophys. J. **92**, 4356-4368. (10.1529/biophysj.106.100032) 17384074 PMC1877768

[36] Kocabey S, Kempter S, List J, Xing Y, Bae W, Schiffels D, Shih WM, Simmel FC, Liedl T. 2015 Membrane-assisted growth of DNA origami nanostructure arrays. ACS Nano **9**, 3530-3539. (10.1021/acsnano.5b00161) 25734977 PMC4415451

[37] Zuraw-Weston SE, Siavashpouri M, Moustaka ME, Gerling T, Dietz H, Fraden S, Ribbe AE, Dinsmore AD. 2021 Membrane remodeling by DNA origami nanorods: experiments exploring the parameter space for vesicle remodeling. Langmuir **37**, 6219-6231. (10.1021/acs.langmuir.1c00416) 33983740

[38] Czogalla A, Kauert DJ, Franquelim HG, Uzunova V, Zhang Y, Seidel R, Schwille P. 2015 Amphipathic DNA origami nanoparticles to scaffold and deform lipid membrane vesicles. Angew. Chem. Int. Ed. **54**, 6501-6505. (10.1002/anie.201501173) 25882792

[39] Franceschi ND, Pezeshkian W, Fragasso A, Bruininks BMH, Tsai S, Marrink SJ, Dekker C. 2022 Synthetic membrane shaper for controlled liposome deformation. ACS Nano **17**, 966-978. (10.1021/acsnano.2c06125) 36441529 PMC9878720

[40] Jahnke K, Huth V, Mersdorf U, Liu N, Goepfrich K. 2022 Bottom-up assembly of DNA cytoskeletons for synthetic cells. ACS Nano **16**, 7233-7241. (10.1021/acsnano.1c10703) 35377150 PMC9134502

[41] Arulkumaran N, Singer M, Howorka S, Burns JR. 2023 Creating complex protocells and prototissues using simple DNA building blocks. Nat. Commun. **14**, 1314. (10.1038/s41467-023-36875-5) 36898984 PMC10006096

[42] Mitchison T, Kirschner M. 1984 Dynamic instability of microtubule growth. Nature **14**, 1314. (10.1038/312237a0) 6504138

[43] Green LN, Subramanian HKK, Mardanlou V, Kim J, Hariadi RF, Franco E. 2019 Autonomous dynamic control of DNA nanostructure self-assembly. Nat. Chem. **11**, 510-520. (10.1038/s41557-019-0251-8) 31011170

[44] Green LN, Amodio A, Subramanian HKK, Ricci F, Franco E. 2017 pH-driven reversible self-assembly of micron-scale DNA scaffolds. Nano Lett. **17**, 7283-7288. (10.1021/acs.nanolett.7b02787) 29182337

[45] Agarwal S, Klocke MA, Pungchai PE, Franco E. 2021 Dynamic self-assembly of compartmentalized DNA nanotubes. Nat. Commun. **12**, 3557. (10.1038/s41467-021-23850-1) 34117248 PMC8196065

[46] Ranallo S, Sorrentino D, Ricci F. 2019 Orthogonal regulation of DNA nanostructure self-assembly and disassembly using antibodies. Nat. Commun. **10**, 5509. (10.1038/s41467-019-13104-6) 31796740 PMC6890650

[47] Zhan P, Jahnke K, Liu N, Göpfrich K. 2022 Functional DNA-based cytoskeletons for synthetic cells. Nat. Chem. **14**, 958-963. (10.1038/s41557-022-00945-w) 35725773 PMC9359917

[48] Gentile S, Grosso ED, Pungchai PE, Franco E, Prins LJ, Ricci F. 2021 Spontaneous reorganization of DNA-based polymers in higher ordered structures fueled by RNA. J. Am. Chem. Soc. **143**, 20 296-20 301. (10.1021/jacs.1c09503) PMC8662731 34843256

[49] Deng J, Walther A. 2021 Autonomous DNA nanostructures instructed by hierarchically concatenated chemical reaction networks. Nat. Commun. **12**, 5132. (10.1038/s41467-021-25450-5) 34446724 PMC8390752

[50] Rossi-Gendron C, Fakih FE, Nakazawa K, Chocron L, Endo M, Sugiyama H, Morel M, Rudiuk S, Baigl D. 2022 Isothermal self-assembly of multicomponent and evolutive DNA nanostructures. ChemRxiv. (10.26434/chemrxiv-2022-12jqs) PMC10656289 37524905

[51] Li R, Bowerman B. 2010 Symmetry breaking in biology. Cold Spring Harb. Perspect. Biol. **2**, a003475. (10.1101/cshperspect.a003475) 20300216 PMC2829966

[52] Mohammed AM, Schulman R. 2013 Directing self-assembly of DNA nanotubes using programmable seeds. Nano Lett. **13**, 4006-4013. (10.1021/nl400881w) 23919535

[53] Barish RD, Schulman R, Rothemund PWK, Winfree E. 2009 An information-bearing seed for nucleating algorithmic self-assembly. Proc. Natl Acad. Sci. USA **106**, 6054-6059. (10.1073/pnas.0808736106) 19321429 PMC2660060

[54] Agrawal DK, Jiang R, Reinhart S, Mohammed AM, Jorgenson TD, Schulman R. 2017 Terminating DNA tile assembly with nanostructured caps. ACS Nano **11**, 9770-9779. (10.1021/acsnano.7b02256) 28901745

[55] Mohammed AM, Šulc P, Zenk J, Schulman R. 2016 Self-assembling DNA nanotubes to connect molecular landmarks. Nat. Nanotechnol. **12**, 312-316. (10.1038/nnano.2016.277) 27992412

[56] Jia S et al. 2021 Growth and site-specific organization of micron-scale biomolecular devices on living mammalian cells. Nat. Commun. **12**, 5729. (10.1038/s41467-021-25890-z) 34593818 PMC8484582

[57] Mohammed AM, Velazquez L, Chisenhall A, Schiffels D, Fygenson DK, Schulman R. 2017 Self-assembly of precisely defined DNA nanotube superstructures using DNA origami seeds. Nanoscale **9**, 522-526. (10.1039/C6NR06983E) 27957574

[58] Schaffter SW, Schneider J, Agrawal DK, Pacella MS, Rothchild E, Murphy T, Schulman R. 2020 Reconfiguring DNA nanotube architectures via selective regulation of terminating structures. ACS Nano **14**, 13 451-13 462. (10.1021/acsnano.0c05340) 33048538

[59] Dhanasekar NN, Li Y, Schulman R. 2022 The ion permeability of DNA nanotube channels. bioRxiv. (10.1101/2022.03.04.482952) PMC13569080 42544780

[60] Li Y, Maffeo C, Joshi H, Aksimentiev A, Ménard B, Schulman R. 2022 Leakless end-to-end transport of small molecules through micron-length DNA nanochannels. Sci. Adv. **8**, eabq4834. (10.1126/sciadv.abq4834) 36070388 PMC9451144

[61] Jahnke K, Illig M, Scheffold M, Tran MP, Mersdorf U, Göpfrich K. In press. DNA origami signaling units transduce chemical and mechanical signals in synthetic cells. Adv. Funct. Mater. (10.1002/adfm.202301176)

[62] Yin P, Yan H, Daniell XG, Turberfield AJ, Reif JH. 2004 A unidirectional DNA walker that moves autonomously along a track. Angew. Chem. **116**, 5014-5019. (10.1002/ange.200460522) 15372637

[63] Shin J-S, Pierce NA. 2004 A synthetic DNA walker for molecular transport. J. Am. Chem. Soc. **126**, 10 834-10 835. (10.1021/ja047543j) 15339155

[64] Wickham SFJ, Bath J, Katsuda Y, Endo M, Hidaka K, Sugiyama H, Turberfield AJ. 2012 A DNA-based molecular motor that can navigate a network of tracks. Nat. Nanotechnol. **7**, 169-173. (10.1038/nnano.2011.253) 22266636

[65] Cha T-G, Pan J, Chen H, Salgado J, Li X, Mao C, Choi JH. 2013 A synthetic DNA motor that transports nanoparticles along carbon nanotubes. Nat. Nanotechnol. **9**, 39-43. (10.1038/nnano.2013.257) 24317284

[66] Yehl K, Mugler A, Vivek S, Liu Y, Zhang Y, Fan M, Weeks ER, Salaita K. 2015 High-speed DNA-based rolling motors powered by RNase H. Nat. Nanotechnol. **11**, 184-190. (10.1038/nnano.2015.259) 26619152 PMC4890967

[67] Blanchard AT, Bazrafshan AS, Yi J, Eisman JT, Yehl KM, Bian T, Mugler A, Salaita K. 2019 Highly polyvalent DNA motors generate 100 + pN of force via autochemophoresis. Nano Lett. **19**, 6977-6986. (10.1021/acs.nanolett.9b02311) 31402671

[68] Bazrafshan A et al. 2021 DNA gold nanoparticle motors demonstrate processive motion with bursts of speed up to 50 nm per second. ACS Nano **15**, 8427-8438. (10.1021/acsnano.0c10658) 33956424

[69] Piranej S, Bazrafshan A, Salaita K. 2022 Chemical-to-mechanical molecular computation using DNA-based motors with onboard logic. Nat. Nanotechnol. **17**, 514-523. (10.1038/s41565-022-01080-w) 35347272 PMC9119907

[70] Bazrafshan A, Meyer TA, Su H, Brockman JM, Blanchard AT, Piranej S, Duan Y, Ke Y, Salaita K. 2020 Tunable DNA origami motors translocate ballistically over μm distances at nm s^−1^ speeds. Angew. Chem. **132**, 9601-9608. (10.1002/ange.201916281) PMC7301628 32017312

[71] Stömmer P, Kiefer H, Kopperger E, Honemann MN, Kube M, Simmel FC, Netz RR, Dietz H. 2021 A synthetic tubular molecular transport system. Nat. Commun. **12**, 4393. (10.1038/s41467-021-24675-8) 34285204 PMC8292359

[72] Ibusuki R, Morishita T, Furuta A, Nakayama S, Yoshio M, Kojima H, Oiwa K, Furuta K. 2022 Programmable molecular transport achieved by engineering protein motors to move on DNA nanotubes. Science **375**, 1159-1164. (10.1126/science.abj5170) 35271337

[73] Kopperger E, List J, Madhira S, Rothfischer F, Lamb DC, Simmel FC. 2018 A self-assembled nanoscale robotic arm controlled by electric fields. Science **359**, 296-301. (10.1126/science.aao4284) 29348232

[74] Ibusuki R, Shiraga M, Furuta A, Yoshio M, Kojima H, Oiwa K, Furuta K. 2020 Collective motility of dynein linear arrays built on DNA nanotubes. Biochem. Biophys. Res. Commun. **523**, 1014-1019. (10.1016/j.bbrc.2019.12.125) 31973818

[75] Endo M, Sugiyama H. 2018 DNA origami nanomachines. Molecules **23**, 1766. (10.3390/molecules23071766) 30022011 PMC6099981

[76] Bath J, Turberfield AJ. 2007 DNA nanomachines. Nat. Nanotechnol. **2**, 275-284. (10.1038/nnano.2007.104) 18654284

[77] Urban MJ, Both S, Zhou C, Kuzyk A, Lindfors K, Weiss T, Liu N. 2018 Gold nanocrystal-mediated sliding of doublet DNA origami filaments. Nat. Commun. **9**, 1454. (10.1038/s41467-018-03882-w) 29654323 PMC5899135

[78] Bertosin E, Maffeo CM, Drexler T, Honemann MN, Aksimentiev A, Dietz H. 2021 A nanoscale reciprocating rotary mechanism with coordinated mobility control. Nat. Commun. **12**, 7138. (10.1038/s41467-021-27230-7) 34880226 PMC8654862

[79] Quail T, Golfier S, Elsner M, Ishihara K, Murugesan V, Renger R, Jülicher F, Brugués J. 2021 Force generation by protein–DNA co-condensation. Nat. Phys. **17**, 1007-1012. (10.1038/s41567-021-01285-1)

[80] Zhan P, Urban MJ, Both S, Duan X, Kuzyk A, Weiss T, Liu N. 2019 DNA-assembled nanoarchitectures with multiple components in regulated and coordinated motion. Sci. Adv. **5**, eaax6023. (10.1126/sciadv.aax6023) 31819901 PMC6884410

[81] Peil A, Xin L, Both S, Shen L, Ke Y, Weiss T, Zhan P, Liu N. 2022 DNA assembly of modular components into a rotary nanodevice. ACS Nano **16**, 5284-5291. (10.1021/acsnano.1c10160) 35286063 PMC9047004

[82] Kuzyk A, Schreiber R, Zhang H, Govorov AO, Liedl T, Liu N. 2014 Reconfigurable 3D plasmonic metamolecules. Nat. Mater. **13**, 862-866. (10.1038/nmat4031) 24997737

[83] Xin L, Duan X, Liu N. 2021 Dimerization and oligomerization of DNA-assembled building blocks for controlled multi-motion in high-order architectures. Nat. Commun. **12**, 3207. (10.1038/s41467-021-23532-y) 34050157 PMC8163789

[84] Göpfrich K, Urban MJ, Frey C, Platzman I, Spatz JP, Liu N. 2020 Dynamic actuation of DNA-assembled plasmonic nanostructures in microfluidic cell-sized compartments. Nano Lett. **20**, 1571-1577. (10.1021/acs.nanolett.9b04217) 32083879 PMC7307956

[85] Ketterer P, Willner EM, Dietz H. 2016 Nanoscale rotary apparatus formed from tight-fitting 3D DNA components. Sci. Adv. **2**, e1501209. (10.1126/sciadv.1501209) 26989778 PMC4788491

[86] Pumm A-K et al. 2022 A DNA origami rotary ratchet motor. Nature **607**, 492-498. (10.1038/s41586-022-04910-y) 35859200 PMC9300469

[87] Shi X et al. 2022 Sustained unidirectional rotation of a self-organized DNA rotor on a nanopore. Nat. Phys. **18**, 1105-1111. (10.1038/s41567-022-01683-z)

[88] Gerling T, Wagenbauer KF, Neuner AM, Dietz H. 2015 Dynamic DNA devices and assemblies formed by shape-complementary, non-base pairing 3D components. Science **347**, 1446-1452. (10.1126/science.aaa5372) 25814577

[89] Illig M, Jahnke K, Scheffold M, Mersdorf U, Drechsler H, Diez S, Göpfrich K. 2023 Self-assembly and contraction of micron-scale DNA rings. bioRxiv. (10.1101/2023.03.09.531887) PMC10940629 38485920

[90] Poppleton E, Mallya A, Dey S, Joseph J, Šulc P. 2021 Nanobase.org: a repository for DNA and RNA nanostructures. Nucleic Acids Res. **50**, D246-D252. (10.1093/nar/gkab1000) PMC8728195 34747480

[91] Franceschi ND, Pezeshkian W, Fragasso A, Bruininks BM, Tsai S, Marrink SJ, Dekker C. 2023 Synthetic membrane shaper for controlled liposome deformation. ACS Nano **17**, 966-978. (10.1021/acsnano.2c06125) PMC9878720 36441529

